# Premature Death of a Schizophrenic Patient due to Evacuation after a Nuclear Disaster in Fukushima

**DOI:** 10.1155/2019/3284153

**Published:** 2019-04-07

**Authors:** Yuki Sonoda, Akihiko Ozaki, Arinobu Hori, Asaka Higuchi, Yuki Shimada, Kana Yamamoto, Tomohiro Morita, Toyoaki Sawano, Claire Leppold, Masaharu Tsubokura

**Affiliations:** ^1^Department of Nursing, Jyoban Hospital, Tokiwa Foundation, Iwaki, Fukushima 972-8322, Japan; ^2^Department of Breast Surgery, Jyoban Hospital, Tokiwa Foundation, Iwaki, Fukushima, 972-8322, Japan; ^3^Hori Mental Clinic, Minamisoma, Fukushima 979-2335, Japan; ^4^Medical Governance Research Institute, Minato-ku, Tokyo 108-0074, Japan; ^5^Department of Surgery, Minamisoma Municipal General Hospital, Minamisoma, Fukushima 975-0033, Japan; ^6^Department of Internal Medicine, Navitas Clinic, Tachikawa, Tokyo, 190-0023, Japan; ^7^Department of Internal Medicine, Soma Central Hospital, Soma, Fukushima 976-0016, Japan; ^8^Global Public Health Unit, School of Social and Political Science, University of Edinburgh, Edinburgh EH8 9LD, UK

## Abstract

Although psychiatric patients are likely to be adversely impacted by disasters, information regarding the processes involved in adverse impacts is limited. In March 2011, Japan experienced an earthquake, tsunami, and the Fukushima Daiichi Nuclear Power Plant accident. In its aftermath, Takano Hospital, 22 km south of the power plant, underwent forced patient evacuation. A 54-year-old Japanese male with schizophrenia, who had been hospitalized in the psychiatric ward for over 20 years, was transferred and experienced a series of hospital relocations. Although his physical status was intact when he left Takano Hospital, his condition gradually worsened, presumably due to incomplete exchange of patient information between institutions and changes in the treatment environment. Having developed ileus a few days prior, he was bedridden when he returned to Takano Hospital in May 2011. Over the course of treatment, he developed aspiration pneumonia and died in August 2011. A review of medical records revealed that all his purgative medicines had been stopped after his evacuation, possibly contributing to the development of ileus. This case highlights the necessity of establishing systems enabling patient information sharing between institutions in disaster settings and the importance of recognizing that long-term evacuation may have fatal impacts for psychiatric patients.

## 1. Introduction

Disasters, such as earthquakes, tsunamis, and hurricanes, can cause various health issues among affected populations. Examples of direct health effects include blunt trauma, crush-related injuries, drowning, and acute infections [1, 2]. Vulnerable populations such as the elderly may be more susceptible to these direct health effects than others owing to cognitive decline, chronic illness, and/or immobility [3–5]. In addition, disasters can lead to indirect health consequences among the vulnerable. Indeed, an increased mortality risk was observed among evacuated hospitalized patients and nursing home residents, compared to those who did not evacuate, during Hurricane Katrina and the Fukushima nuclear crisis [6–9]. Considering that the occurrence of disasters is currently on the rise [10], it is becoming increasingly important to establish concrete measures to mitigate their direct and indirect health effects in vulnerable populations.

It has recently been suggested that in the wake of disasters, there is a higher risk of morbidity and mortality among psychiatric patients as compared to the general population [11]. Possible reasons for this are that, similar to other vulnerable populations such as the elderly, psychiatric patients are likely to experience direct health consequences owing to difficulties in responding to rapidly changing postdisaster conditions and carrying out appropriate self-protective behaviors [12]. However, information on the ways psychiatric patients may be impacted by indirect health effects of disasters is limited.

**Figure 1:**
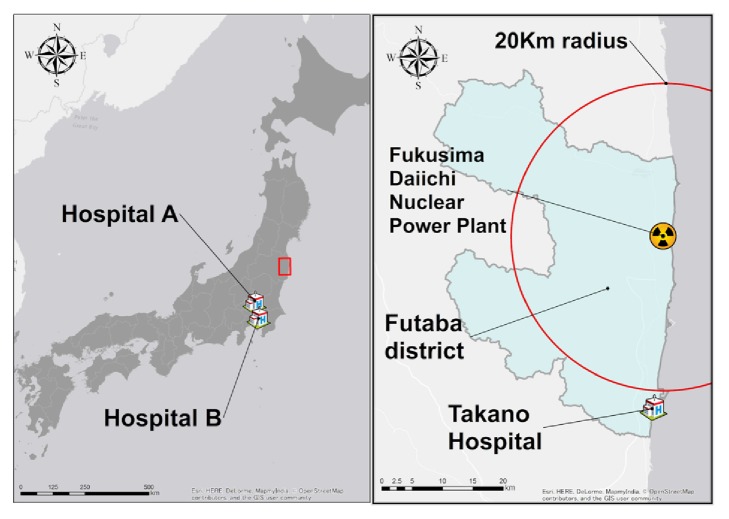
Map of Takano Hospital, Hospital A, and Hospital B. Takano Hospital is located 22 km south of Fukushima Daiichi Nuclear Power Plant. Hospitals A and B are located 250 km and 255 km south of Takano Hospital.

The Fukushima Daiichi Nuclear Power Plant (FDNPP) accident, which occurred following the earthquake and tsunami on March 11, 2011, led to nearly all local residents being forced to evacuate from Futaba District (Figure 1), which housed the FDNPP [13]. Although five hospitals with a total of 901 psychiatric beds originally operated in the district, all were forced to close and evacuate their patients immediately following the disaster [14]. Takano Hospital (Figure 1), located 22 km south of the FDNPP, was no exception. The director of Takano Hospital had originally chosen to retain all patients in an attempt to avoid exposing bedridden and critically ill patients to the risks of forced evacuation immediately after the disaster; however, he eventually had to relocate the physically stable, including most psychiatric patients, to decrease the burden on the limited number of staff who remained with him and save medical resources for those who needed them the most in the weeks following the disaster [15].

Here, we report the case of a 54-year-old male with a history of schizophrenia who experienced a series of forced evacuations and died five months following the 2011 triple disaster. This case shows the devastating indirect health impacts of forced evacuation procedures on psychiatric patients following large-scale disasters.

## 2. Case Presentation

This is the case of a 53-year-old Japanese male with schizophrenia who had been hospitalized at Takano Hospital from the age of 29. Haloperidol was continuously prescribed, and he was mentally stable before the disaster. His parents died during his hospitalization, and he did not have any social or financial support from other family members, which was one reason for his long-term hospitalization. This type of long-term hospitalization of psychiatric patients is relatively common in Japan [16]. His right eyesight and hearing were impaired. He had chronic constipation and took purgative medicines, yet no other abnormalities were noted in terms of his physical condition. Although he maintained positive relationships with the hospital staff, few people regularly visited him, and he had scarce contact with the outside world.

From March 11, 2011, the day of the earthquake and tsunami, to March 19, 2011, the staff at Takano Hospital provided consistent care to the patient, and his mental and physical condition did not significantly deteriorate despite the chaotic postdisaster situation. On March 19, 2011, the number of staff who remained at the hospital dropped to 13, compared to the predisaster baseline of 88. Following the hospital director's decision to evacuate relatively stable patients, the patient in question was transferred to Hospital A (Figure 1), which specializes in psychiatric care, in Saitama Prefecture, 250 km away from Takano Hospital, together with 36 other patients on a microbus. Although a doctor, nurses, and hospital clerks accompanied the patients during this bus ride, some patients developed dehydration because of limited water intake and long hours of driving. (There was no specific information available about how our patient coped with the bus ride.) After the transfer, the staff of Takano Hospital handed over each patient's paper-based chart to the health workers at Hospital A. Although it is common for health workers in Japan to write notes summarizing patients' conditions and treatments to hand over to new facilities upon patient transfer, in the present case, the Takano Hospital staff were not able to write summary notes owing to the increased workload and decreased manpower after the disaster. After admission to Hospital A, although no specific disease development was noted and the patient did not complain of symptoms or ask for help, his general condition progressively worsened, and he needed increasingly more assistance in all aspects of daily living (i.e., waking, eating, excretion, and bathing) from the staff. On April 27, 2011, the staff found that he had a bloody stool, and he was transferred to Hospital B (Figure 1), a tertiary center in Tokyo, for endoscopic examinations and intensive care. The staff at Hospital A summarized his condition and treatment in their facility and handed over this document to Hospital B. All purgative medicines were stopped at Hospital B, and esophagogastroduodenoscopy (EGD) and colonoscopy (CS) were conducted on April 28 and May 9, 2011, respectively, yet they failed to detect any abnormalities. Although his bloody stools spontaneously stopped while at Hospital B, he developed ileus due to severe constipation and progressively weakened and lost independence. His purgative medicines were not resumed. He was transferred back to Takano Hospital on May 11, 2011.

He was completely bedridden when readmitted to Takano Hospital. Although his mental condition was reported to be stable throughout the evacuation period, it had become more difficult for the staff at Takano Hospital to communicate with him. Once his meals were stopped owing to his ileus, his abdominal condition began to improve. Health workers resumed his oral intake of meals on May 24, 2011, yet his appetite was not completely restored. He developed a fever on June 30, and on July 1, chest computed tomography revealed aspiration pneumonia and pleural effusion. Although his pneumonia was treated with antibiotics, his condition consistently deteriorated, and he died on August 16, 2011, at the age of 53.

## 3. Discussion

This is the case of a hospitalized patient with schizophrenia who experienced forced evacuation and died following the 2011 Fukushima nuclear crisis. This case suggests that postdisaster forced evacuation procedures can lead to significant indirect health effects, as well as potentially fatal outcomes, for hospitalized psychiatric patients.

Long-term evacuation may result in substantial stress on psychiatric patients. It has been suggested that patients with schizophrenia may not be able to express their physical complaints as easily as the general population [12]. Resultantly, detection of life-threatening conditions, such as cancer or acute abdomen, can be delayed [17]. The patient in question did not complain about his progressively worsening condition at Hospital A. It has additionally been reported that psychiatric patients are vulnerable to abrupt changes in their environment [12]. Although the health workers at Takano Hospital established a rapport with the patient during his long-term hospitalization, upon relocation to new facilities, he was in unfamiliar environments and might not have been able to ask for help easily. As such, in conjunction with communication difficulties due to his psychiatric disorder and hearing and eyesight impairment, long-term evacuation and multiple transfers may have put him in a difficult situation, leading to significant stress.

In cases of prolonged evacuation and transfers from one facility to another, it can be challenging to share patient information between facilities and individual health workers. In this particular case, all laxatives were discontinued at Hospital B. Given that the patient had taken these drugs for a long period, this was an event that may have triggered his ileus and eventual development of aspiration pneumonia and death only five months after the disaster. In general, antipsychotics are prescribed to psychiatric patients on the long term, and laxatives are concomitantly used to combat representative side effects, such as chronic constipation [18]. Therefore, discontinuation of laxatives can cause suppression of intestinal peristalsis, leading to the development of ileus among psychiatric patients. Vomiting during ileus may have led to aspiration pneumonia in the present case. It is true that stopping the purgative medication was necessary for the emergency endoscopic tests (EGD and CS). However, if the importance of the patient's continuous receipt of laxatives had been communicated to Hospital B, the interruption of the medication may have been limited to a minimum period. We speculate that it may have been quite difficult for Hospital A to report all relevant information about this case to Hospital B upon transfer, as his hospitalization period since the first admission to Takano Hospital was quite long.

According to the Japanese Civil Code, hospitals have no responsibility regarding follow-up of patients in the period between evacuation from the hospital and returning.

While hospitals are obliged to make arrangements for patients to receive appropriate medical treatment in general settings, this obligation does not apply to emergencies such as disasters. To the best of our knowledge, no litigation regarding the violation of obligation to protect patients' safety was reported despite the fact that high mortality rates were observed among patients in hospitals and nursing care facilities during the evacuations after the accident at the FDNPP. However, in order to protect the health of vulnerable people in the event of a disaster, it is important to establish patient information sharing systems that can function after disaster, allowing health care providers to understand the health conditions of patients and evacuees at any medical facility as easily as possible. To achieve this, relevant stakeholders—local health workers, public health professionals, and politicians—should recognize that disaster-affected patients may experience multiple relocations, and that it can be difficult to successfully share patient information from one hospital to another, as shown in this case.

One possible solution is to describe key points for patient management in easy-to-understand medical records during regular medical examination and to periodically create and update summaries that reflect prescription, psychiatric diagnosis, activities of daily living, presence or absence of intellectual impairment, family, and social services. The utilization of these medical records may not only enable health providers to share information in general settings, but also achieve decreased mortality and morbidity among vulnerable populations, including psychiatric patients, in disaster settings.

The other possible solution is the development of cloud servers, which maintain all medical records in one database [19, 20]. Such systems could enable health care providers to share patient information in general settings and to achieve decreased mortality and morbidity among vulnerable populations, including psychiatric patients, in disaster settings. However, as this solution may entail additional costs and effort, its utility will require verification.

## 4. Conclusion

This case suggests that forced evacuation following disasters can have detrimental health effects on patients with psychiatric disorders, with potentially fatal consequences. Communication difficulties and vulnerability to changes in environment, specific to psychiatric patients, as well as the difficulties of patient information sharing, a universal issue in postdisaster settings, may contribute to the adverse health effects of disasters on psychiatric patients. There is a need for a system by which multiple medical facilities can share patient information in the wake of disasters so as to reduce unnecessary changes in treatment and further disruption in the lives of already vulnerable patients.
